# Spatial Generalization in Operant Learning: Lessons from Professional Basketball

**DOI:** 10.1371/journal.pcbi.1003623

**Published:** 2014-05-22

**Authors:** Tal Neiman, Yonatan Loewenstein

**Affiliations:** 1 Edmond and Lily Safra Center for Brain Sciences, The Hebrew University, Jerusalem, Israel; 2 Department of Neurobiology, The Alexander Silberman Institute of Life Sciences, Department of Cognitive Science and Center for the Study of Rationality, The Hebrew University, Jerusalem, Israel; Oxford University, United Kingdom

## Abstract

In operant learning, behaviors are reinforced or inhibited in response to the consequences of similar actions taken in the past. However, because in natural environments the “same” situation never recurs, it is essential for the learner to decide what “similar” is so that he can generalize from experience in one state of the world to future actions in different states of the world. The computational principles underlying this generalization are poorly understood, in particular because natural environments are typically too complex to study quantitatively. In this paper we study the principles underlying generalization in operant learning of professional basketball players. In particular, we utilize detailed information about the spatial organization of shot locations to study how players adapt their attacking strategy in real time according to recent events in the game. To quantify this learning, we study how a make \ miss from one location in the court affects the probabilities of shooting from different locations. We show that generalization is not a spatially-local process, nor is governed by the difficulty of the shot. Rather, to a first approximation, players use a simplified binary representation of the court into 2 pt and 3 pt zones. This result indicates that rather than using low-level features, generalization is determined by high-level cognitive processes that incorporate the abstract rules of the game.

## Author Summary

According to the law of effect, formulated a century ago by Edward Thorndike, actions which are rewarded in a particular situation are more likely to be executed when that *same* situation recurs. However, in natural settings the *same* situation never recurs and therefore, generalization from one state of the world to other states is an essential part of the process of learning. In this paper we utilize basketball statistics to study the computational principles underlying generalization in operant learning of professional basketball players. We show that players are more likely to attempt a field goal from the vicinity of a previously made shot than they are from the vicinity of a missed shot, as expected from the law of effect. However, the outcome of a shot can also affect the likelihood of attempting another shot at a different location. Using hierarchical clustering we characterize the spatial pattern of generalization and show that generalization is primarily determined by the *type* of shot, 3 pt vs. 2 pt. This result indicates that rather than using low-level features, generalization is determined by high-level cognitive processes that incorporate the abstract rules of the game.

## Introduction

*Of several responses made to the* ***same*** *situation, those which are accompanied or closely followed by satisfaction to the animal will … be more firmly connected with the situation, so that, when it* ***recurs*** *, they will be more likely to* ***recur*** *…’* (Edward Thorndike, 1874–1949) [1].

*No man ever steps into the* ***same*** *river twice, for it's not the* ***same*** *river and he's not the* ***same*** *man* (Heraclitus of Ephesus, 535–475 BCE) [2].

Humans and animals modify their behavior in response to the consequences of their previous actions, a process known as operant learning. The standard account for this learning is based on a family of reinforcement learning (RL) algorithms that assert that the computational problem of learning from experience is achieved through the synergy of two processes: first, the *values* of the different actions (or more generally, state-actions) are learned from past actions and their subsequent rewards; second, these learned values are used to choose (or to learn to choose) among different actions such that actions associated with a higher values are more likely to be chosen [3]–[5] but see also [6]–[8]. This account is based, to a large extent, on a large number of laboratory experiments, in which participants repeatedly choose between the same small number of alternative actions (e.g., press a button) in repeated settings and are rewarded according to these actions.

By contrast, in many natural environments, organisms learn from the consequences of their past actions in settings in which the same situation and action never recur (not even in the sense that two “identical” trials “recur” in a laboratory experiment). In these cases, generalization is an essential part of operant learning [9]. In this process of generalization, the organism determines which past situations, actions and their consequences are relevant for the current situation. In the language of RL algorithms discussed above, generalization is the process of determining which set of different situations defines a state and which set of responses defines an action. The level of generalization determines, roughly speaking, the density parsing of the set of situations into states and the set of responses into actions. A limited generalization would result in a large number of state and actions in the process of learning whereas broad generalization would result in a small number of states and actions. Too limited generalization implies that the organism learns values of states that are essentially identical, resulting in too-slow learning. Too broad generalization implies that the organism is inferring the outcome of future responses from irrelevant past experience, which may lead to suboptimal behavior even after very long learning. Thus, the proper level of generalization, which determines the tradeoff between the speed and the accuracy of learning, is of an utmost importance in the process of learning. It should be noted that the question of the proper level of generalization is present even in RL models that assume continuous states and actions [10], [11].

The problem of determining the proper level of generalization is not limited to operant learning. Indeed, this question has received considerable attention in the framework of Pavlovian learning and supervised learning (see [12] and references within). The goal of this paper is to elucidate the cognitive strategy underlying generalization in operant learning in natural conditions.

Professional basketball, which is played by highly motivated and extensively-trained players, provides an exceptional opportunity to quantitatively study generalization in operant learning in complex natural environments. The objective of players in basketball is to gain points by shooting a ball through a hoop. If successful, the team is awarded with two or three points, depending on the distance of the shot attempt from the basket. In a previous study we demonstrated that players modify their shot selection policy in response to the recent history of their shots and their outcomes [13]. After a made (successful) 3-point (3 pt) shot, the probability of attempting another 3 pt shot is 30% higher than that probability after a missed 3 pt. Moreover, some of the variability in players' shot selection can be accounted for using standard RL algorithms. However, lacking additional information about the shots, our previous study was unable to address the question of what is considered by the players as “the same situation” and “the same action”.

Consider a player in possession of the ball. Multiple factors, including the locations, velocities and postures of team and opponent players, the score and the time in the game are relevant to the decision of whether or not to attempt a field goal (FG). In the framework of RL, all these factors determine the state of the world. In this paper we focus on the spatial location of the payers, which provides us with a low dimensional projection of the state of the world at the time of the FG. Quantifying how the outcome of a FG in one spatial location affects subsequent FGs in different locations is thus informative about the pattern and level of generalization between states. A spatially restricted generalization implies that the outcome of shots made in a particular location would have very little effect of behavior in other locations of the court. By contrast, learning could be independent of shot location, implying substantial spatial generalization. Between these two extremes, a made shot in one location may enhance the probability of another shot from the vicinity of that location, but not from further away locations. Alternatively, the pattern of generalization may be more complex. For example, a made shot in one location may enhance the probability of another shot from the same distance, the same angle or from the symmetrical location relative to the basket. Identifying the patterns of spatial generalization is thus the objective of this study.

## Results

### The spatial organization of field goal attempts

**Figure 1:**
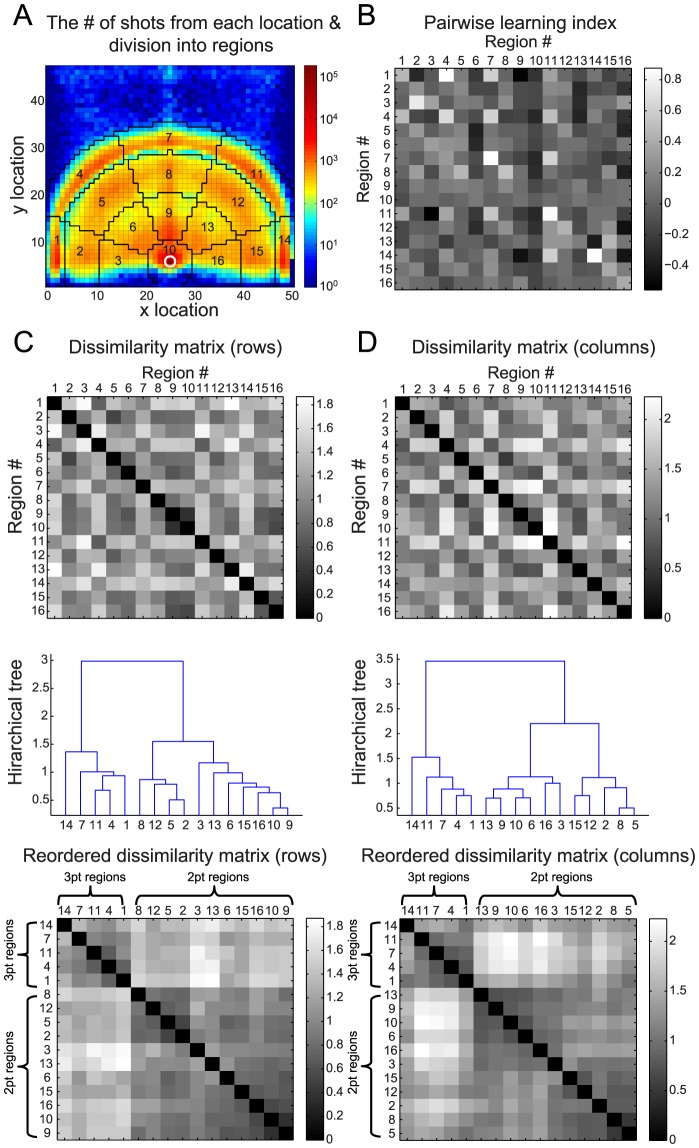
The spatial organization of learning. **A**. The spatial distribution of all 759,050 FGs in our dataset. The basket is depicted by a white circle and the upper boundary is the half court line. The color codes for the number of FGs taken from each location in 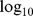 scale. The black lines delineate 16 regions used in subsequent analysis. **B**. The averaged learning matrix 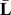, based on 161,302 FGs attempted by 166 players that passed our selection criteria (see Materials and Methods). **C**. Top, Dissimilarity matrix, 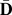, computed based on the rows of the matrix 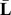 in **B** such that 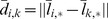 is the Euclidian distance between the rows 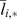 and 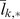 of 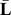; Middle, Hierarchical clustering of the matrix in **B** based on the dissimilarity between the rows (see Material and Methods); Bottom, the dissimilarity matrix ordered according to the dendrogram in the middle panel. **D**. same as in **C** for the columns of matrix 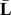.

We examined the records of all players from the National Basketball Association (NBA) in four regular seasons and considered their 759,050FGs, measured at a 1×1 ft^2^ resolution. The spatial distribution of FGs is presented in Figure 1A, which depicts the two-dimensional histogram of the FGs locations, pooled from all players. The white circle denotes the location of the basket and the upper boundary is at the half-court line. The color codes for the number of shots attempted from each location in a logarithmic scale. As shown in Fig. 1A, the distribution of shot locations is not homogeneous. Rather, there are islets of higher FG probability. In our analysis, we used these islets to define 16 regions, delineated by black lines in Fig. 1A.

In order to quantify how the outcome of a FG attempted from region 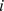 affects subsequent behavior at region 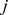 we computed, for each player, three probabilities: the a-priori probability that a player would attempt a FG from region 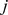, 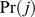, and two conditional probabilities: the probabilities that a player would attempt a FG from 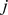, given that his previous FG was a made or missed FG from region 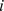, 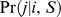 and 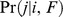 respectively (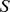 and 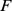 denote Successful and Failed FG). These three probabilities determine a learning matrix 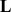 whose entries are given by
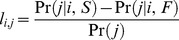
(1)To gain insight into 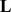, we consider a player that incorporates a fixed policy that is insensitive to the outcome of past FGs, i.e., a player that does not learn from past made and missed FGs. In this case, because behavior is independent of the outcome of the previous FG, the two conditional probabilities are equal, 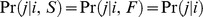. As a result, 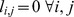. Alternatively, consider the extreme case in which a player is very sensitive to the outcome of the previous FG: after a made FG he always attempts another FG from the same region whereas the FG immediately following a miss FG is never repeated from the same region. In this case, 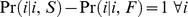 and 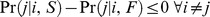. In other words, all the diagonal elements of 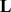 are positive and all off-diagonal elements are non-positive. More generally, 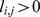 for two regions 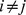 implies a generalization from region 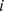 to region 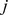: A made shot in region 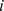 motivates subsequent FG attempts from region 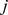 whereas a missed shot in 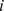 discourages FG attempts from 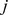. Therefore the learning matrix 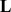 is informative about the generalization pattern in learning.

We computed the matrix 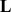 for all players who passed our selection criterion (166 players, 161,302 FGs, see Materials and Methods). The matrix 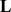, averaged over all players, denoted by 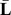, is depicted in Fig. 1B. Several points are noteworthy when considering 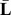. First, the diagonal elements of 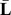 tend to be positive (14/16 diagonal elements in 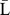 are positive, p<0.003, one-tailed binomial test). This implies that a made FG motivates players to attempt another FG from the same region relative to a missed FG. To quantify this tendency to repeat successful actions and to avoid unsuccessful ones, we considered the mean value of the diagonal terms: 
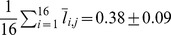
. Roughly speaking, this average implies that on average, the outcome of a FG changes the probability that a FG will be repeated from the same region by approximately 38%. Second point worthwhile noting is that many of the off-diagonal elements are also positive and that the magnitude of some of them is substantial. For example, the largest off-diagonal element 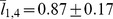 is as large as the largest diagonal element, 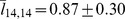. However, not all off-diagonal terms are positive. For example, while a made shot in region 1 almost doubles the likelihood of a shot in region 4 compared to a missed FG (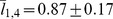), it substantially decreases the likelihood of attempting another shot from region 9 (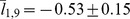) and has almost no effect on the likelihood that the next shot will be from region 8 (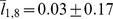). To quantify this heterogeneity in the values of the off-diagonal terms, we computed the standard deviation of the distribution of the off-diagonal elements and found that 
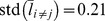
. This number, which is significantly larger than the expected standard deviation in a process, in which transitions between regions on successive FGs are random (*p*<0.001 Monte Carlo permutation test, see Materials and Methods) is a measure of the spatial heterogeneity in the generalization: different regions differ by approximately 21% in their response to made and missed FGs in the other regions.

### Clustering analysis

To better understand the pattern of generalization depicted in the matrix 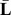 (Fig. 1B), we note that the 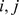 element in 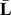, 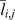, is a measure of the effect of the outcome of the shot from region 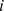 on the likelihood that the subsequent shot would be from region 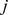. Therefore, the *row* 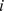 of the matrix 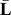, denoted as 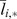, is a measure of the effect of the outcome of a FG attempts in region 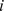 on all subsequent FG attempts. If two rows of the matrix 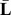 are similar, 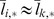, then the outcome of FGs in regions 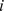 and 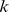 similarly affect subsequent behavior. By contrast, if these two rows are very different then we can infer that made and missed FGs from these two regions are treated differently in the process of learning. Therefore, the similarity between the rows of the matrix 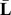 is a measure of the pattern of generalization in the learning.

To study the similarity between the rows, we computed the dissimilarity matrix 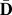, where 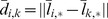 is the Euclidian distance between rows 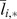 and 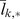 of 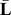 (Fig. 1C, Top). To identify the regions that similarly affect subsequent behavior we constructed a hierarchical tree (dendrogram) of the rows of 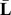 (Fig. 1C, Middle) using agglomerative hierarchical clustering (Materials and Methods). The dissimilarity matrix, reordered according to the hierarchical tree is presented in Fig. 1C (Bottom). As clearly seen in Figs. 1C Middle and Bottom, regions 1, 4, 7, 11 and 14 (left branch in Fig. 1C, Middle) are grouped together in the clustering analysis. Interestingly, this grouping follows the separation into 3 pt regions (areas 1, 4, 7, 11 and 14) and 2 pt regions (all other regions). It should be noted that this grouping into 3 pt and 2 pt regions is not spatially local (e.g., regions 1 and 14 are furthest apart). Rather, it reflects the distance from the hoop. Further considering the finer clustering structure, we find that in the 2 pt branch (right branch in Fig. 1C, Middle), regions 2, 5, 8 and 12 are grouped together and are separated from the other 2 pt regions. This grouping contains all long-distance 2 pt regions except one (region 15) and none of the short-distance 2 pt regions.

The clustering analysis of Fig. 1C was aimed at finding sets of regions that “*affect*” all other regions in a similar way. However, there is a complementary way of defining patterns of generalization. We can consider which regions are similarly “*affected*” by the outcomes of FGs in all other regions. The former analysis (Fig. 1C) is based on *prospective* similarity, whereas the latter analysis is based on *retrospective* similarity. Formally, prospective clustering is based on similarity between the *rows* of 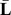 whereas retrospective clustering is based on similarity between the *columns* of 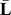. Because the matrix 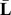 is not symmetrical, prospective and retrospective clustering are not identical and in principle may yield different patterns of clustering. Thus, we repeated the clustering analysis for the columns of 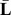 (Fig. 1D).The results of this analysis are similar to those of the prospective clustering. The most prominent separation of the regions is into 3 pt and 2 pt regions (Fig. 1D, Middle). Moreover, within the 2 pt branch (right branch in Fig. 1D, Middle), the long-distance 2 pt regions (2, 5, 8, 12 and 15) are also clustered together and separately from the shorter-distance 2 pt regions.

In summary, (1) the prospective and the retrospective clustering yielded similar findings; (2) To a first approximation, learning is dominated by the separation of FGs into 2 pt and 3 pt shots, a grouping that is not spatially local. (3) To a lesser extent, the 2 pt FGs are further clustered into two groups, short-distance and long-distance 2 pt FGs. It is interesting to note that the clustering analysis did not reveal any evidence of generalization that is based on the angle of the shooting player from the rim.

Next, we further studied how the outcome of a FG attempt in one region affects subsequent attempts in other regions. Because the analysis depicted in Fig. 1 indicates that to a first approximation players cluster the spatial locations into three regions, we used in our subsequent analysis a coarser partition of the court into three regions: 3 pt FG attempts (areas 1, 4, 7, 11 and 14), long-distance 2 pt FG attempts (2, 5, 8, 12 and 15) and short-distance 2 pt FG attempts (all other regions).

Similar to the analysis of Fig. 1B, we computed for each player the learning matrix corresponding to this coarser division of the court, 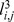, where 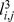 is defined as 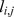 (Eq. 1) such that the three regions 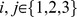 correspond to 3 pt regions, the long-distance 2 pt regions and the short-distance 2 pt regions, respectively. Averaging over the players yields the 3×3 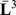 learning matrix
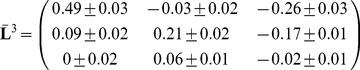
(2)Where each entry denotes the value of 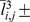
standard error of the mean (SEM). Several points are noteworthy. First, 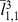 is by far the largest element, indicating that made and missed FGs in the 3 pt region primarily affect subsequent 3 pt attempts such that a made 3 pt increases the likelihood of another FG from that region and a missed FG decreased it. This is consistent with our previous study, in which we have demonstrated that the probability of a 3 pt attempt increases after a made 3 pt and decreases after a missed 3 pt [13]. Second, the two clusters of 2 pt FGs are differentially affected by the 3 pt FG attempts. Short-distance, but not long-distance 2 pt are sensitive to the outcome of the previous 3 pt (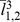 and 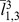, respectively). Third, 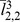 is positive and large, indicating that players tend to repeat a long-distance 2 pt if made, and to avoid it if missed. This change of policy comes primarily at the expense of the short-distance 2 pt. Fourth, long-distance 2 pt FG attempts have a positive albeit small effect on 3 pt FGs, such that a made long-distance 2 pt increases the probability of a 3 pt attempt (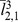). Finally, made and missed short-distance 2 pt FGs have only a small effect on subsequent FGs (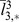).

### Distance analysis

The results presented in Fig. 1 suggest that in the process of learning, players reduce the complexity of the environment by treating the outcome of FG attempts made at different locations as if they were from the same location. The clustering analysis indicates that this generalization is primarily determined by the distance of the FG attempt from the basket. To better understand how the distance from the basket affects learning, we reanalyzed the spatial pattern of generalization with a finer distance resolution, at the expense of angular information, using to the following procedure: for each player, we binned all FGs according to their distance from the basket at a 2 ft resolution, separately for 2 pt and 3 pt FGs. For each bin, we separated the FGs according to their outcome, made or miss, and separately computed, for each of these outcomes, the conditional probability that the next FG would be a 3 pt FG. The difference between these two conditional probabilities is a measure of the dependence of the magnitude of operant learning on the distance of the FG from the basket. Note that this focus on the difference in conditional probabilities of a 3 pt FGs as a measure of learning, rather than on the distribution of locations of the following FGs as in Fig. 1, is motivated by our finding presented in the previous section that the outcomes of FGs primarily affect the probability of a 3 pt FG (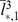 in Eq. 2). This focus on a scalar learning variable for each distance, rather than a vector, enabled us to study learning at a substantial finer spatial resolution than we could if we have focused on a learning vector, as in Fig. 1.

**Figure 2:**
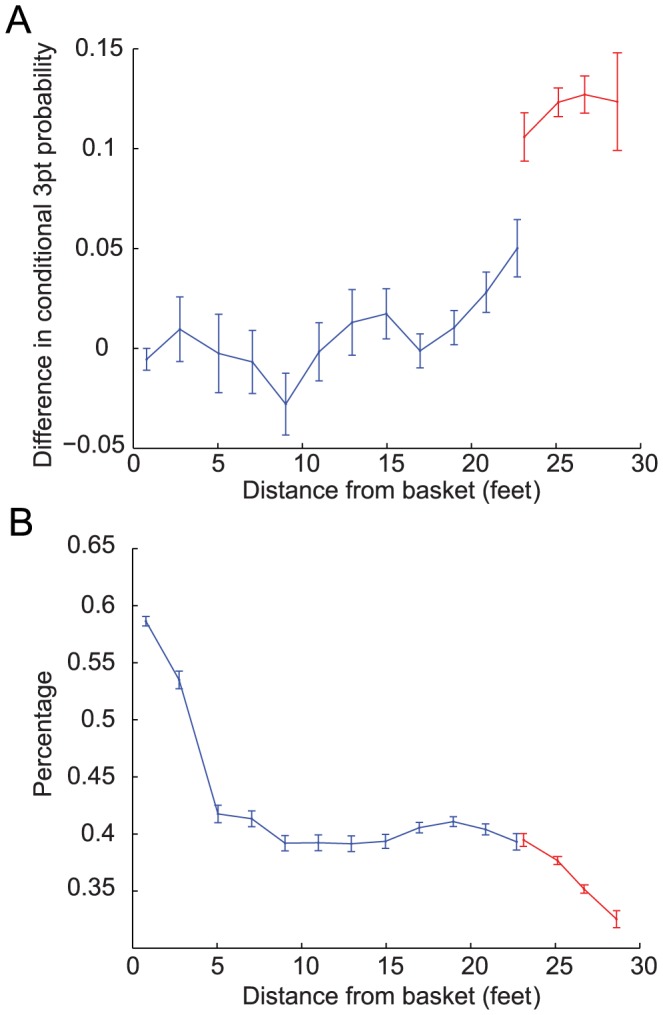
The effect of distance from the basket on learning. **A**. the difference between the probabilities of attempting a 3 pt after a make and after a miss FG as a function of the distance of the first shot from the basket. FGs were sorted into 2ft wide bins according to their distance from the basket. For each bin and for each player, we calculated the average distance from the basket and the conditional probabilities and then averaged over the players. **B**. The shooting percentage as a function of the distance from the basket. The percentage is defined as the ratio between the number of made FGs and the number of attempted FGs. Error bars denote the SEM. The blue dots denote 2 pt FGs and the red dots denote 3 pt FGs. Analysis is based on 263,557 FGs of 300 players that passed our selection criteria (see Materials and Methods).

The difference between the conditional probabilities, averaged over all players that passed our selection criterion (300 players, see Material and Methods), is depicted in Fig. 2A, where the blue and red dots depict 2 pt and 3 pt bins, respectively. We find that the effect of the outcome of a FG on the probability that the following FG would be a 3 pt FG increases with the distance of the FG from the basket. This results is in agreement with the finding of the previous section that 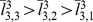. However remarkably, the increase in the probability is not continuous. Rather, there is a marked discontinuity in the magnitude of learning when comparing 2 pt and 3 pt FG bins. To further quantify this discontinuity, we used the fact the 3 pt line that separates the 2 pt and 3 pt regions is not equidistant from the basket. Near the corners of the court, the 3 pt line is closer to the basket than near the center. Therefore, whether or not a FG made at a distance between 22 ft and 23.75 ft is a 2 pt or 3 pt FG is determined by the angle to the FG relative to the basket. This enables us to dissociate the effect of distance on learning from the effect of the identity of the FGA on learning. As depicted in Fig. 2A, the leftmost red dot in Fig. 2A and the rightmost blue dot in Fig. 2A correspond to 3 pt and 2 pt FGs attempted at almost identical distance from the basket (23.1 ft and 22.7 ft, respectively). Nevertheless, the difference in the magnitudes of learning, quantified as the differences in the conditional probabilities, is substantial and significant (0.11±0.01 and 0.05±0.01 for the 3 pt and 2 pt FGs, respectively, *p*<0.001 Monte Carlo permutation test). This discontinuity in the learning magnitudes entails that the abstract classification of a FG as a 2 pt or 3 pt is an important aspect of the generalization. In other words, with respect to learning, players learn from the outcome of 2 pt and 3 pt FGs in a categorically different manner, even if these FGs were attempted from the same distance from the basket. These results imply that rather than low-level features such as the physical distance, reasoning which is based on the abstract rules of the game, dominate the pattern of generalization.

Does the pattern of generalization reflect the difficulty of the FG? Naively, one could argue that the more distant a FG is, the more difficult it is and therefore the more informative a made FG is about the current capabilities of the player (and/or the abilities of the opponent players). Therefore, a made long-distance FG influences subsequent FGs more than a made FG short-distance FG. According to this view, 3 pt FGs are more difficult than 2 pt FGs. Therefore, they are more informative and thus have a larger effect on subsequent FGs. Moreover, because there is a categorical difference in payoff associated with 2 pt and 3 pt FGs, the defense team is likely to be more motivated to prevent made 3 pt FGs than to prevent made 2 pt FGs. As a result, 3 pt FGs may be better guarded and thus categorically more difficult than 2 pt FGs. Such discontinuity in the difficulty could result in a discontinuity in the learning magnitude in the transition from 2 pt to 3 pt FGs, depicted in Fig. 2A. In order to test this hypothesis, we computed the shooting percentage of FGs from different distances for the same 300 players analyzed in Fig. 2A. The shooting percentage is the ratio of made FGs and attempted FGs, and thus is a measure of the difficulty of the FG. The average shooting percentage as a function of the distance is depicted in Fig. 2B. As predicted, the shooting percentage decreases with the distance from the basket. However, the dependence of the percentage on the distance does not closely follow the dependence of the learning signal on the distance. In particular, the shooting percentages of 2 pt and 3 pt FGs from the same distance are 0.393±0.007 (rightmost blue dot) and 0.395±0.006 (leftmost red dot), respectively, which are not significantly different from each other (*p*>0.16, Monte Carlo permutation test). Thus, a difference between the difficulties of the 2 pt and 3 pt FGs cannot account for the discontinuity in the magnitude of learning. This result indicates that it is the identity of the shot as a 2 pt or 3 pt shot per se, and not the difficulty of the FG, that plays the dominant role in the players' pattern of generalization.

### Basketball and the matching law

In psychology and neuroscience, there is a long tradition of foraging-like experiments, in which the subject, human or animal, repeatedly chooses between a small number of alternatives and is rewarded according to his choices. In many of these experiments, the probability that a choice would be rewarded decreases with the frequency of choosing that alternative, corresponding to a situation known as “diminishing return” in economics. Interestingly, the aggregate behavior in many of these experiments follows a behavioral regularity known as “the “matching law”: the fraction of reward accumulated from choosing an action is proportional to the fraction of times the action was chosen [14]–[17]. Put differently, subjects allocate their choices such that the average reward per choice is equal for all chosen alternatives. Interestingly, the matching law is widespread despite the fact that in many cases, it does not correspond to the policy that maximizes the average reward. Therefore, the computational principles underlying this law of behavior have been a subject of discussion for decades (see [18] and references therein). According to the Theory of Melioration put forward by Herrnstein and Prelec [19], subjects estimate the return from the different alternatives and shift their choice preference in the direction of the alternatives that provide a higher-than-average return (see [7], [8], [20]–[22] for a neural implementation of this algorithm). In a diminishing return reward schedule, the shift in choice preference in favor of an alternative reduces the return from that alternative. This dynamical learning process reaches a fixed point when choices are allocated such that the return from all chosen alternatives is equal.

**Figure 3:**
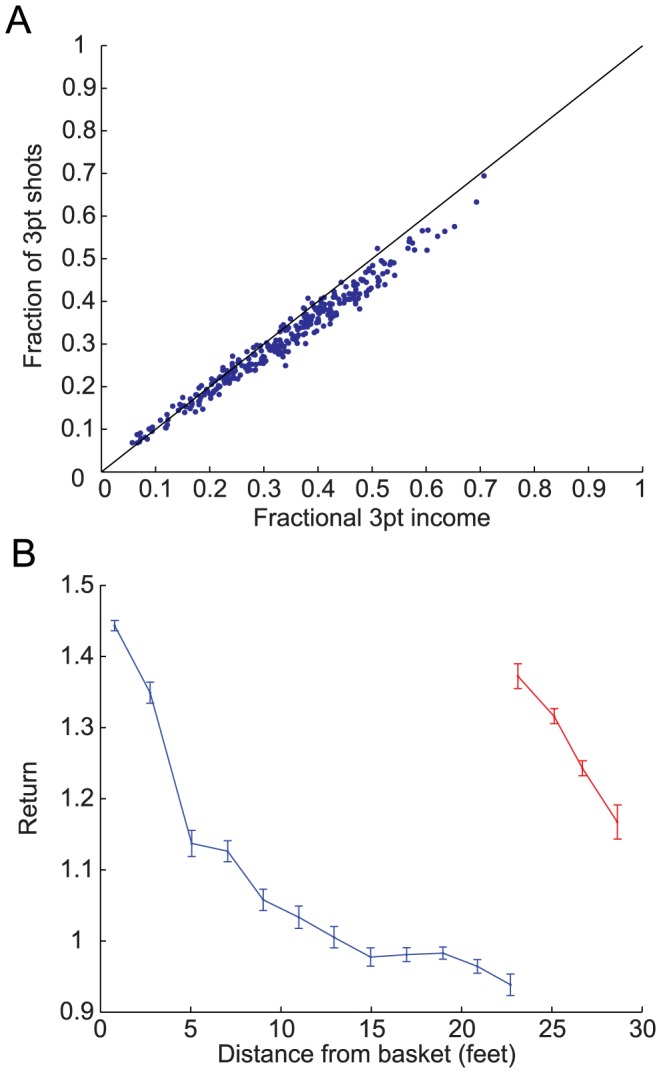
Matching and deviations from matching. **A**. The fraction of 3 pt FG attempts as function of the fractional 3 pt income, defined as the fraction of points gained by the offensive team from the time of the 3 pt shot until the time that the opposing team got hold of the ball. Each point corresponds to a single player that passed our selection criterion (same as in Fig. 2, see Materials and Methods) and the black diagonal line denotes the behavior expected from the matching law. **B**. The return as a function of the distance from the basket for 2 pt (blue) and 3 pt (red) FGs. The matching law predicts that the returns would be equal. Each point is an average over all players in A and error bars are SEM.

Previous studies have reported that basketball players' allocation of 2 pt and 3 pt FGs approximately conforms to the matching law: the fraction of 3 pt attempts matches the fraction of points gained by 3 pt shots [23]–[25]). This is demonstrated in Fig. 3A. Each circle in Fig. 3A corresponds to the fraction of 3 pt shots as a function of the fraction of points gained from 3 pt FGs for a single NBA player (see Materials and Methods). The diagonal line corresponds to the behavior predicted by the matching law.

While the “reward schedule” in basketball is far more complex and far less accessible than the reward schedule used in standard operant learning experiments, “diminishing return” with the frequency of attempting a FG from a spatial location is likely to play a role. The reason is that increasing the frequency of attempts from a location may be associated with the player attempting more difficult FGs, e.g. FGs that are better guarded by the defensive team. Moreover, adaptive defensive maneuvers will also contribute to the diminishing of return. Therefore, the matching of the returns associated with the 2 pt and 3 pt FGs could indicate that the basketball players meliorate. What are the state-actions in the process of melioration? If the spatial generalization is restricted and the number of effective states is large then the 2 pt and 3 pt matching could result from matching of a large number of actions, each associated with a different state, all of which are endowed with approximately the same return. Alternatively, generalization may be substantial and in that case matching is a macroscopic phenomenon, associated with the separation of states into 2 pt and 3 pt regions but microscopically, at a higher resolution, the matching law is not followed. Thus, the spatial distribution of returns is indicative of the level of generalization.

To test this, we used the results presented in Fig. 2B to compute the return of FGs as a function of the distance of the FG from the basket. As is illustrated in Fig. 3B, despite the fact that the aggregate returns of 3 pt and 2 pt FGs are comparable (1.314±0.007 and 1.173±0.004, respectively) when computing the returns on a finer spatial resolution substantial deviations from the matching law are observed. This result further substantiate the finding that it is abstract classification of the shot as a 2 pt or 3 pt shot dominates the players' pattern of generalization.

## Discussion

In this study we used professional basketball to probe the cognitive strategy underlying generalization in operant learning in natural conditions. Generalization in learning indicates that events taking place in one state of the world affect the policy utilized in other states of the world. We used the spatial locations of players at the times of shot attempts as a proxy for the state of the world, and quantified the level of generalization between the states. Using hierarchical clustering analysis of the spatial conditional probabilities of shots we found that the pattern of generalization indicates that the separation of FGs into 2 pt and 3 pt shots, high-level abstract features of the game that seem of little relevance to the learning task, dominates the pattern of generalization. This result is also supported by the finding that while the matching law is a good approximation to the aggregate allocation of FGs into 2 pt and 3 pt shots, there are substantial deviations from this law of behavior on a finer spatial resolution.

We interpret these findings as resulting from learning processes that are taking place in the brains of the shooting players. However, because these players operate in a complex strategic setting, other contributors to behavior should be considered. For example, the outcome of the shot may affect defensive maneuvers, which could change the spatial distribution of subsequent shots. However, this mechanism is unlikely to be a main contributor because defense is likely to respond to a made shot by preventing another shot from the same location, whereas we found that the diagonal elements of 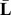 tend to be positive. Another potential contributor to the changes observed in behavior may be changes in the behavior of the player's coach or teammates. In a previous study we discussed this possibility at length. In short, if changes in a player's behavior result from actions taken by the player's coach or teammates then the learning observed in players within the same team are expected to be correlated. However, such correlations were not found [13]. Thus we conclude that while it is likely that many additional factors contribute to behavior, it is learning processes within the shooting players that dominate the generalization patterns reported in this paper.

Models of operant learning often take as given that the learner has full information about the relevant states and actions in the problem. However, in real-life situations (and probably also in laboratory settings), the necessary preliminary step of identifying these states and actions is an essential part of operant learning. This is also often the case in machine learning, where classification is often preceded by feature extraction [26]. Our results highlight the importance of this preprocessing stage in the learning [9], [27].

Another contribution of this work is methodological. The distinction between the prospective and retrospective similarity may prove beneficial when studying generalization in other learning tasks. For example, in a standard supervised learning paradigm, the participant is instructed to learn the mapping between 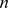 stimuli 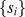 and their desired responses 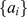. The learning is quantified by computing the set of probabilities 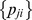 that denote the probability that the response of the participant to stimulus 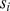, is  [28]. These probabilities can be used to define a 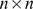 matrix whose entries are 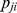. The prospective and retrospective clustering analysis that we used to study generalization in operant learning can be readily applied to this task to better quantify the pattern of generalization.

Recent years have seen a growing interest in data acquired from professional sports. Basketball data, in particular, were used to study various phenomena, such as decision making in shot selection [13], [29]–[33], the ‘hot hand’ belief [34], [35], how coaching experience affects the effectiveness of timeouts [36], and the dynamics of scoring within a game [37]. Off the court, basketball players were subjects in imaging experiments examining the underlying neural mechanisms of action anticipation and evaluation [38], [39]. A major advantage of professional basketball is that large quantities of carefully collected behavioral data can be used to study the behavior of highly-motivated and extensively-trained humans in their natural settings, complementing the more controlled experiments in laboratory settings. With the development of high-speed cameras and automatic image processing, the extensive public interest in sports' statistics can be utilized to enhance our understanding of the computational principles underlying different cognitive processes.

## Materials and Methods

All individual games data in the form of play-by-play is available online. (http://www.basketballgeek.com/data/).

### Data analysis and statistical procedures

Regions 1–16 defined in Fig. 1A contained 2.9%, 5.6%, 4.2%, 5.7%, 3.3%, 3.3%, 6.9%, 5.5%, 6.9%, 3.8%, 2.4%, 4.1%, 5.0%, 4.0%, 2.4% and 33.3% of the FGs, respectively, such that less than 1% of the FGs were attempted from outside these 16 regions and thus were excluded from the analysis. The criterion for including a player at the analysis presented in Fig. 1B–D was that he attempted at least 10 FG from each of the 16 regions in the season. The number of players that met this criterion was 167, where a player is defined per season (e.g., if the same player passed our criteria in two seasons he is counted twice). However, one of the players was discarded because one of the rows in the learning matrix 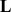 was ill-defined.

In the analysis of Fig. 2, each player's FGs were sorted into 2 ft wide bins according to their distances from the hoop. A player was included in this analysis if he missed at least one FG and made at least one FG from each of the bins, a criterion which resulted in 300 players.

All statistical analyses are within-player: the numbers were computed separately for each player and then were averaged over the players, giving equal weight to each player in the average; averages reported are accompanied by the SEM. In addition, all analyses are within a game and therefore, in all conditional probabilities we only considered the effect of the outcome of shots 1 to *N*-1 on the locations of shots 2 to *N*, where *N* is the index of the last shot made by the player in the game.

When quantifying the heterogeneity in the values of the off-diagonal term of Fig. 1B we computed the standard deviation of the distribution of the off-diagonal terms. In order to show that this standard deviation is larger than expected by chance assuming no learning, we performed the following Monte Carlo permutation test: independently for each player, we estimated the prior probability of a FG in each of the 16 regions. Then, we computed the pair-wise learning index (Eq. 1) for a surrogate data in which the subsequent FG was replaced with a FG drawn from the estimated of the prior probability. By averaging over all players we obtained a substitute 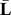 matrix in which there is no generalization between successive FGs. The reported *p*-value indicates the number of times out of 10^4^ repetitions of this procedure in which the standard deviation obtained from surrogate data exceeded the standard deviation of the original data.

Similar tests were performed when testing for significant between the rightmost blue bin and leftmost red bin in Fig. 2A and 2B. For each player included in that analysis, we estimated the prior conditional 3 pt probabilities and percentages from the two bins. Then, we computed the difference in the conditional 3 pt probabilities and the percentage for surrogate data drawn from the estimation of the prior probabilities and percentages, and averaged over the players. The reported *p*-value indicates the number of times out of 10^4^ repetitions of this procedure in which the statistic of the surrogate data exceeded the statistic of the original data.

For the analysis in Fig. 3 we define the return of an FG to be the number of points gained by the team of the shooting player from the time of the FG until the opposing team got hold of the ball. The analysis is based on the data of the 300 players used in Fig. 2. For each player we computed the return of every FG.

### Hierarchical clustering

For each player that passed our criteria, we used Eq. 1 to compute the pair-wise learning index between every 2 regions delineated in Fig. 1A. The results were averaged across the players to form a matrix, 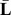 (Fig. 1B), whose 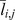 entry measures how much (on average) made and missed FGs from region 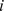 are generalized to region 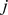. We performed agglomerative hierarchical clustering [40] on 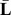 (Fig. 1C) and on 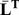 (Fig. 1D). The initial clustering consisted of 16 clusters, one of for each row of 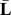 (and 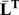). At each step of the algorithm, the two clusters with the lowest distance between them are merged to form one cluster. We used Ward's linkage to measure the distance between clusters, such that the distance between clusters 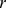 and 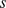 is given by:
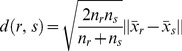
(3)Where 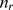 and 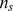 are the number of elements in clusters 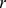 and 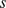 respectively, the norm is the Euclidian distance and 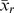 and 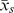 are the centroids of these clusters where the centroid of a cluster 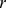 is defined as 
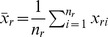
 where 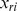 is the 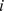-th object in the cluster.
